# Behavior Change Strategies in Digital Exercise Interventions for Adolescent Idiopathic Scoliosis: Scoping Review

**DOI:** 10.2196/66981

**Published:** 2025-09-16

**Authors:** Yufeng Li, Fangyuan Chang, Wen Zhang, Zihan Ren, Yawen Chen, Zhao Liu

**Affiliations:** 1 School of Design Shanghai Jiao Tong University Shanghai China; 2 Department of Spine Surgery Shandong Provincial Hospital Affiliated to Shandong First Medical University Jinan China

**Keywords:** adolescent idiopathic scoliosis, physical rehabilitation, digital technology, digital intervention, behavior change strategy, spine, adolescents, scoping review, exercise therapy, Theoretical Domains Framework, motivation

## Abstract

**Background:**

Adolescent idiopathic scoliosis is a common spinal deformity typically treated with exercise therapy. Despite the increasing use of digital technologies in interventions, there remains a gap in understanding how to effectively integrate behavior change techniques (BCTs) and behavior theories within these digital solutions.

**Objective:**

This review aims to identify the digital characteristics of interventions and the BCTs used, and to analyze potential theoretical mechanisms with the Theoretical Domains Framework and the capability, opportunity, motivation, and behavior model.

**Methods:**

We conducted a scoping review according to the PRISMA-ScR (Preferred Reporting Items for Systematic Reviews and Meta-Analyses extension for Scoping Reviews) guidelines. A total of 5 databases, including PubMed, Web of Science, Embase, Cochrane Library, and CINAHL, were selected for screening eligible studies up to April 4, 2024. We included studies of any design type that involved patients with adolescent idiopathic scoliosis using digital interventions for exercise rehabilitation, including qualitative, quantitative, or mixed methods studies, and study protocols with detailed descriptions of digital interventions. Two researchers independently screened studies and extracted data into tables for descriptive analysis. The Mixed Methods Appraisal Tool was used to assess the quality of studies.

**Results:**

Out of the 3267 identified papers, 21 (0.64%) studies were included. The most frequently used technologies were videoconferencing (n=7) and instructional videos (n=5). The three most common BCT clusters were “Shaping Knowledge” (n=19), “Social Support” (n=16), and “Antecedents” (n=16). “Knowledge” was the most used mechanism of action (n=21), followed by “Skills” (n=16), “Environmental Context and Resources” (n=16), and “Social Influences” (n=16). The studies primarily addressed “Capability” and “Opportunity,” with less emphasis on “Motivation,” particularly “Automatic Motivation.”

**Conclusions:**

This review identified common digital technologies and their characteristics, analyzed potential mechanisms of behavior change in interventions, and provided recommendations for technology utilization. Future research should further evaluate the effectiveness of digital technologies while enhancing patient motivation and user experience.

**Trial Registration:**

PROSPERO CRD42024530851; https://www.crd.york.ac.uk/PROSPERO/view/CRD42024530851

## Introduction

Adolescent idiopathic scoliosis (AIS) is a 3D spinal deformity [1,2] that primarily affects adolescents aged 10 to 18 years [3]. AIS has an overall prevalence of 0.47%-5.2% and is more common in girls, with a female-to-male ratio ranging from 1.5:1 to 3:1, which increases with age and curve severity [4,5]. It can result in cosmetic deformities, respiratory dysfunction, functional limitations, and negative impacts on quality of life and mental health [3,6]. Exercise therapy, including general therapeutic exercises (GTE) and physiotherapeutic scoliosis–specific exercises (PSSE), is an effective nonsurgical treatment for mild to moderate scoliosis, particularly for patients who are still in their growth phase with lower Risser grades. It can slow curve progression and improve quality of life as a standalone therapy [7-10]. For patients with more severe curves, it serves as an adjunct to bracing to enhance treatment effectiveness and minimize brace-related side effects [9]. However, it is typically administered in clinical settings, requiring intensive physiotherapist supervision, long-term treatment, and repeated in-person engagement, which imposes a financial burden on families and increases the workload of health care professionals [11,12].

Digital technologies are increasingly used in exercise therapy, either as standalone interventions or as components of broader interventions, to address the limitations of traditional therapy [13-16]. Digital interventions offer a promising solution for promoting scoliosis rehabilitation, particularly home-based care. For instance, telerehabilitation technologies enable remote interactions between patients and physiotherapists, improving cost-effectiveness and accessibility [13,17]. Additionally, virtual reality (VR) can simulate treatment environments, enhancing patient engagement and rehabilitation outcomes [15]. However, the lack of physiotherapist supervision in digital interventions can lead to reduced adherence and improper exercise performance [18]. This issue primarily relates to patient behavior. For patients with AIS, starting and maintaining a prescribed exercise regimen requires behavior change, influenced by motivation and various internal and external factors [19-22]. Without external support, such as physiotherapist supervision, patients often struggle to achieve and sustain this change independently [23]. For example, a randomized trial in AIS found that a home exercise group supported by an instructional DVD achieved 67% compliance, compared to 95% in a physiotherapist-supervised group, along with a higher dropout rate (35% vs 11%) and poorer outcomes [24]. Similarly, a nonrandomized trial comparing clinic-based and telemedicine-delivered yoga for AIS reported lower adherence in the remote group (63% vs 80%) and slower curve improvement [25]. In addition to the lack of external support, factors such as exercise complexity and patients’ beliefs about the benefits of training significantly influence adherence [26]. These challenges reveal a critical gap: without effective support for sustained engagement, home-based digital rehabilitation may fall short of its intended outcomes. Moreover, they underscore the need for a more systematic understanding of how digital tools are experienced and used in real-world rehabilitation settings.

Theories are fundamental to behavior change interventions and help identify the behavior change strategies required [27,28]. Using established theoretical frameworks to describe interventions is also essential for understanding their effectiveness and informing future intervention design [23]. Thus, incorporating behavior change strategies, including behavior change techniques (BCTs) and behavioral theories, into digital intervention development is particularly important. This integration helps identify target behaviors, promotes desired changes, and achieves expected health outcomes, particularly for unsupervised home rehabilitation [16,27,29,30]. The inherent support mechanisms of digital technologies can also shape, modify, or strengthen user behaviors, thereby facilitating behavior change [16,31,32]. For example, posture management programs based on the theory of planned behavior have been shown to improve cognitive outcomes and physical conditions in patients with AIS [33]. Another study found that certain elements of behavior change strategies, such as peer-based training and the use of assistive devices, may improve scoliosis rehabilitation [26]. However, to date, no review has summarized the progress of digital interventions in AIS exercise therapy, resulting in a limited understanding of their effectiveness in supporting patient behavior change. At the theoretical level, many existing interventions lack a theoretical foundation, and few studies explain how specific features of digital interventions influence rehabilitation behaviors. As a result, the theoretical pathways between intervention components and behavioral outcomes remain unclear. Moreover, evidence suggests that interventions based on a single theory may improve behavioral intentions, but are often less effective in achieving actual behavior change [34].

To address these gaps, this scoping review identifies the BCTs used and maps them to corresponding Theoretical Domains Framework (TDF) domains and capability, opportunity, motivation, and behavior (COM-B) components. It further assesses the characteristics and potential theoretical mechanisms of existing interventions, thereby promoting the development of theory-driven and evidence-based digital solutions [35-39]. Specifically, by applying the Behavior Change Technique Taxonomy v1 (BCTTv1) developed by Michie et al [35], in combination with the TDF, researchers can identify specific BCTs within interventions and map them to corresponding TDF domains. This enables a detailed analysis of intervention content alongside the underlying theoretical mechanisms. This approach has been widely applied in health intervention research, particularly in the context of chronic diseases [27,35,40,41]. Furthermore, adopting integrated, multitheoretical frameworks allows for a more comprehensive understanding of target behaviors [27,34,41,42]. This review adopts both the COM-B model and the TDF, two widely used frameworks for identifying behavioral determinants and developing targeted interventions [36,37,43]. The COM-B model, central to the Behavior Change Wheel, identifies three essential elements for performing a target behavior: capability, opportunity, and motivation [37]. The TDF provides a broader understanding of behavior, with 14 domains mapped onto the COM-B model [36,44,45].

This scoping review aims to summarize existing literature on digital interventions for AIS exercise therapy to address the knowledge gap. This review included the following objectives: (1) characterize digital interventions and user experiences; (2) identify the theoretical foundations of interventions, and determine the BCTs used and their corresponding TDF domains and COM-B model components; and (3) synthesize common theoretical frameworks and potential mechanisms of action from existing studies, and provide recommendations for future digital intervention development.

## Methods

### Protocol and Registration

This study was a scoping review that adhered to the PRISMA-ScR (Preferred Reporting Items for Systematic Reviews and Meta-Analyses extension for Scoping Reviews) guidelines [46]. The completed PRISMA-ScR checklist is provided in Multimedia Appendix 1. An a priori protocol was developed and registered on PROSPERO (CRD42024530851).

This review was initially registered with PROSPERO as a systematic review. Upon further consideration, we adopted a scoping review approach, as our aim was to explore and synthesize existing evidence on the use of digital technologies in AIS exercise rehabilitation and to identify key features and research gaps. A scoping review is particularly suitable for emerging topics and provides a broad conceptual overview of existing evidence [47]. Accordingly, we adapted the search strategy, data extraction, and analysis methods to align with the objectives of this scoping review. The inclusion and exclusion criteria for this scoping review are provided in Textbox 1.

**Eligibility criteria.**

**Inclusion criteria**

- Studies targeting patients with adolescent idiopathic scoliosis (AIS) or including them
- Interventions that are digital or incorporate a digital component
- Digital interventions supporting exercise rehabilitation
- Patients with access to digital apps
- Studies of any design type, including qualitative, quantitative, or mixed methods studies, and study protocols with detailed descriptions of digital interventions
- Studies written in English.
- Studies published in peer-reviewed journals with full text available

**Exclusion criteria**

- Studies not involving patients with AIS
- Patients with orthopedic, neurological, or psychiatric conditions that hinder exercise
- Recent or planned spinal surgery within 12 months
- Nondigital interventions
- Digital interventions not applied to exercise rehabilitation
- Conference abstracts, commentaries, reviews, or books
- Duplicate reports of the same study

### Information Sources and Search Strategy

Searches were conducted from the inception of the databases up to April 4, 2024, across the following five electronic databases: PubMed (National Library of Medicine), Web of Science (Clarivate Analytics), Embase (Elsevier), Cochrane Library (Wiley), and CINAHL (EBSCO), without any restrictions. The electronic search strategy (Multimedia Appendix 2) was developed and executed under the guidance of an experienced librarian. The search terms (Table 1) covered three main topics and their subtopics: AIS, exercise therapy, and digital intervention. Additional sources were identified through manual searches, including reviewing the reference lists of studies included in this scoping review to minimize the chances of missing relevant studies.

**Table 1:** Search terms.

Topics	MeSH^a^ terms	Free-text terms
Adolescent idiopathic scoliosis	“Scoliosis”	“Scolioses” OR “Idiopathic Scoliosis” OR “Adolescent Idiopathic Scoliosis” OR “Juvenile Idiopathic Scoliosis”
Exercise therapy	“Exercise Therapy”	“Remedial Exercise*” OR “Exercise Therapies” OR “Rehabilitation Exercise*” OR “Exercise*” OR “Training” OR “Sport*” OR “Conservative Treatment*” OR “Conservative Intervention*” OR “Physiothera*” OR “Physical Activit*” OR “Physical Therap*” OR “Schroth” OR “SEAS” OR “DoboMed” OR “Side Shift” OR “Lyon” OR “BSPTS” OR “FITS” OR “Stabilization”
Digital intervention	“Digital Health” OR “Telemedicine” OR “Mobile Applications” OR “Internet-Based Intervention”	“Digital Health Technolog*” OR “Digital Intervention*” OR “Digital” OR “Wearable Device*” OR “Wearable Sensor*” OR “Exergaming*” OR “Exergame*” OR “Virtual Reality” OR “Video Game*” OR “Gamification” OR “Telehealth” OR “eHealth” OR “Mobile Health” OR “mHealth” OR “Telenursing” OR “Telerehabilitation” OR “Mobile Application” OR “App” OR “Application*” OR “Smartphone” OR “Smart-Phone” OR “Telephone” OR “Cellphone” OR “Mobile” OR “Email” OR “E-Mail” OR “Tablet” OR “Cell” OR “Computer-Assisted” OR “Computer” OR “Web” OR “Website” OR “Online” OR “Internet” OR “Social Media”

^a^MeSH: Medical Subject Headings.

### Selection of Sources of Evidence

All electronic search records were stored in EndNote X9 (Clarivate) and deduplicated. Two authors (YL and FC) independently screened an initial set of 30 studies and compared their results to ensure a consistent understanding of the screening criteria. Subsequently, they independently screened the remaining records based on titles, abstracts, and full texts. Any discrepancies were resolved through discussion with a third author (ZR). Reasons for full-text exclusions were documented. Additionally, we manually screened the reference sections of eligible full-text sources to identify further relevant studies.

### Data Charting Process

Data charting was collaboratively completed by two authors (YL and FC) using Excel (Microsoft Corp) and was pilot-tested and calibrated within the team. The data extraction table was developed based on the TIDieR (Template for Intervention Description and Replication) checklist [48]. Additionally, we referred to the data charting tool developed by Gooch et al [16], which extends the TIDieR checklist by incorporating elements specific to digital interventions and behavior change methods, aligning with this review. Data coding was independently performed by two authors (YL and FC), with discrepancies resolved through discussion. If consensus could not be reached, a third author (ZR) acted as an adjudicator. Quantitative data extracted from the included studies were coded into predefined categories, and qualitative author statements were also coded. The classification of digital technologies was initially based on prior reviews [16,49] and was expanded as new types of technologies were identified during data extraction. Conceptually related technologies were further grouped into broader thematic clusters. For example, “videoconferencing,” “instructional videos,” and “mobile applications” were categorized under the thematic cluster “Digital Content and Platforms.” Other charted variables (eg, intervention location and duration) were also synthesized into thematic categories by analyzing associations among the initial codes and summarizing them based on the most frequently reported features.

For extracting behavior change strategies in digital interventions, we first coded each study using the BCTTv1 and recorded the frequency of BCT use [35]. Next, based on expert consensus from prior studies linking BCTs to TDF domains in health interventions, and team consensus, the BCTs were mapped onto the TDF mechanisms of action [41,50,51]. Finally, the TDF domains were linked to the COM-B components. The Behavior Change Wheel is a guide for designing interventions, providing explicit connections between TDF domains and COM-B components [45].

### Data Items

Data items related to digital technologies and behavior change strategies are summarized in Table 2. The complete data extraction table is provided in Multimedia Appendix 3 [12,17,24,25,52-68]. In studies where digital technologies were integrated into broader interventions, it was sometimes difficult to isolate the effects of individual components. Consequently, behavior change strategies were coded for the entire intervention.

**Table 2:** Data items in this scoping review.

Data items	Details
Bibliographic Information	Authors, publication year, country, funding statement
Study details	Study design, objectives, research focus
Population details	Study population, sample size, age
Digital intervention details (based on the TIDieR^a^ Checklist)	Digital technology characteristics, user experience, and behavior change strategies
Study outcomes	Outcome measures, results
Evidence gaps	Limitations, future research directions

^a^TIDieR: Template for Intervention Description and Replication.

### Quality Assessment

The quality of the included studies was assessed using the Mixed Methods Appraisal Tool (MMAT) [69,70]. The MMAT is a validated tool that enables the evaluation of the methodological quality of five study categories: qualitative research, randomized controlled trials (RCTs), nonrandomized studies, quantitative descriptive studies, and mixed methods studies. In addition to two general screening questions applicable to all study types, each study design includes five specific questions relevant to its methodology, each scored as “yes,” “no,” or “cannot tell.”

Two authors (YL and FC) independently assessed all studies. Discrepancies were resolved through discussion, and a third author (ZR) was consulted when consensus could not be reached. In line with the aim of this scoping review, no studies were excluded based on the quality assessment results [71].

### Synthesis of Results

Quantitative data were synthesized in Excel using descriptive statistics (eg, frequencies) and presented in tables and figures. Qualitative data on user experiences with digital interventions, including direct user quotes and authors’ interpretations, were synthesized narratively into common themes. Two authors (YL and FC) independently extracted the qualitative data and conducted open coding. Initial codes were discussed and grouped into broader categories, eventually resulting in four main themes. These themes were refined through team discussions to ensure reliability and consensus. Meanwhile, a summary and descriptive analysis were conducted on the general characteristics of included studies, digital technology characteristics, user experiences, and behavior change strategies. To further analyze the use of BCTs across studies and compare them with other reviews, BCTs were classified into 16 BCT clusters for analysis [16,72].

## Results

### Selection of Sources of Evidence

**Figure 1:**
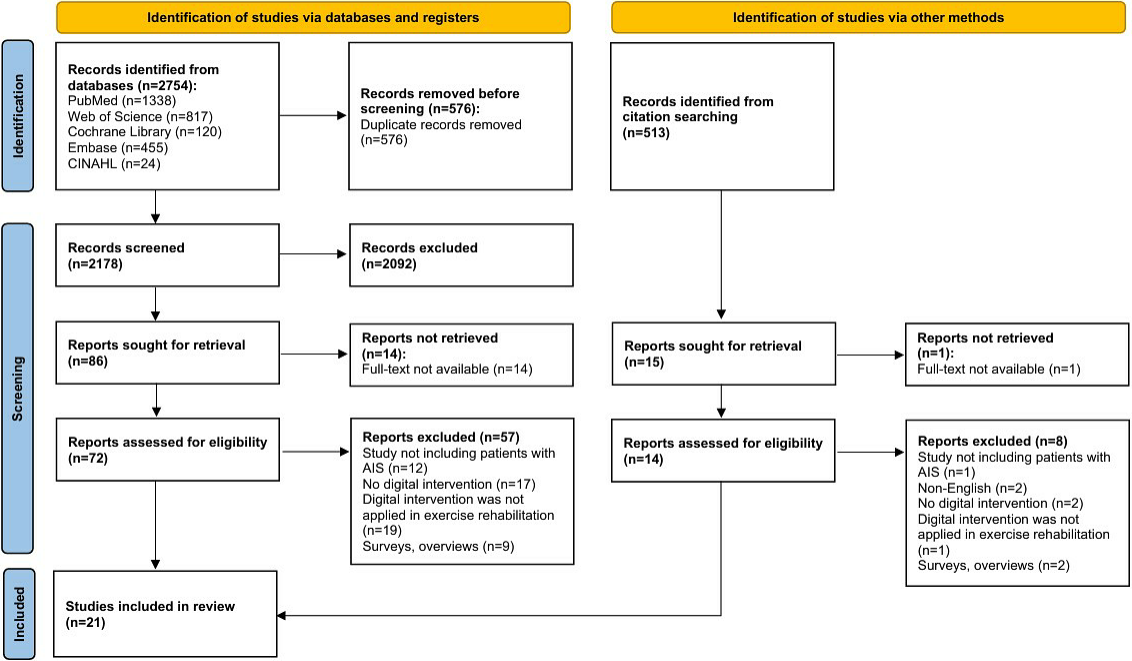
PRISMA (Preferred Reporting Items for Systematic Reviews and Meta-Analyses) flow diagram of the study selection process. AIS: adolescent idiopathic scoliosis.

A total of 3267 studies were identified, including 2754 from databases and 513 from other sources. After removing duplicates and screening titles, abstracts, and full texts, 21 (0.64%) studies were included in the review (Figure 1).

### Description of the Studies

#### Study Characteristics

The studies included in this review were published between 2012 and 2024 (Table 3). After 2020, the publication frequency increased significantly, with most studies (15/21, 71%) published between 2021 and 2024. In total, 4 (19%) studies were conducted in China, followed by the United States (3/21, 14%), Turkey (3/21, 14%), Italy (2/21, 10%), Austria (2/21, 10%), and Brazil (2/21, 10%). Only 1 (5%) study involved multiple countries.

In terms of study design, nearly half (9/21, 43%) were feasibility or pilot studies. Other study designs included RCTs (5/21, 24%), case studies (2/21, 10%), RCT protocols (2/21, 10%), cohort studies (1/21, 5%), non-RCTs (1/21, 5%), and case series (1/21, 5%).

The interventions used various exercise methods. A total of 11 (52%) studies explicitly based their exercise methods on PSSE, encompassing multiple different schools of PSSE [11]. Among these, 7 (33%) studies used the Schroth method. A total of 10 (48%) studies involved GTE.

**Table 3:** Population and study characteristics.

Study	Year	Country	Study design	Population	Age (y)	Sample size	Type of exercise	Digital form
Feistritzer-Gröbl et al [53]	2012	Austria	Case study	AIS^a^	12	1	Schroth exercises	A computer game using a motion-sensing game controller
Sardini et al [54]	2012	Italy	Feasibility or pilot studies	Scoliosis	N/A^b^	No	Generic correction exercises	Wearable sensor
Zapata et al [24]	2015	United States	RCT^c^	AIS with LBP^d^	10-17	34	Spinal stabilization exercises	DVD containing exercise videos
Wibmer et al [55]	2016	Austria	Case series	AIS	7-13	8	Schroth exercises	A video game using a motion-sensing game controller
Liang et al [56]	2018	China	RCT protocol	AIS	10-16	42	3D integrated exercise	WeChat app
Caviedes et al [52]	2020	United States	Feasibility or pilot studies	Scoliosis or LBP	22-64	6 (1 scoliosis, 5 healthy)	Generic correction exercises	Wearable sensor array
Cozeta Anca et al [57]	2021	Romania	Feasibility or pilot studies	AIS	11-17	10	Schroth exercises	Videoconferencing software
Fishman [25]	2021	United States	Non-RCT	AIS	Under 21	56	Specific yoga poses	Videoconferencing software
Lau et al [12]	2021	China	RCT	AIS	11-14	40	7-minute HIIT^e^	Web-based software providing exercise videos
Marin et al [58]	2021	Italy	Feasibility or pilot studies	AIS	11-19	18	Auto correction exercises	Google Meet videoconferencing software or exercise videos on a YouTube channel
Rösner et al [59]	2021	Germany	Feasibility or pilot studies	IS^f^	13-32	9	Hippotherapy	VR^g^ system comprising mechatronic device, therapist GUI^h^, and synchronized visualization
Wang et al [60]	2021	China	RCT protocol	AIS	10-18	40	Schroth exercises and SEAS^i^ exercises	WeChat app
Li et al [61]	2022	China	Feasibility or pilot studies	AIS	N/A	21 (12 students, 9 coaches)	Schroth exercises	Wearable system with training games
Moraes et al [62]	2022	Brazil	Feasibility or pilot studies	AIS	11-13	22 (students)	GPR^j^ postures	VR system comprising a wearable helmet, infrared tracked controllers, and Wii Balance Board
Romano et al [63]	2022	Israel, Italy	Feasibility or pilot studies	RTT^k^ with scoliosis	3.8-38.3	20	Specific physical therapy	Videoconferencing software
Nam et al [64]	2023	South Korea	Feasibility or pilot studies	AIS	14-39	13 (3 AIS, 10 healthy)	Generic correction exercises	Smart-Bar Device and AR^l^ app
Vagner and Bendikova [65]	2023	Czech Republic	Case study	AIS	16	1	Acral coactivation therapy	Video recordings of therapy sessions
Kisa et al [66]	2024	Turkey	RCT	JIA^m^ with AIS	8-16	50	Schroth exercise or core stabilization exercises	WhatsApp videoconferencing software
Andrade et al [67]	2024	Brazil	Cohort study	AIS	10-17	66	Specific exercise program	WhatsApp videoconferencing software
Dursun et al [68]	2024	Turkey	RCT	AIS	10-18	32	Pilates-based exercises	Videoconferencing software
Tombak et al [17]	2024	Turkey	RCT	AIS	10-16	37	Schroth exercises	Video recordings of the exercise process

^a^AIS: adolescent idiopathic scoliosis.

^b^Not applicable.

^c^RCT: randomized controlled trial.

^d^LBP: low back pain.

^e^HIIT: high-intensity interval training.

^f^IS: idiopathic scoliosis.

^g^VR: virtual reality.

^h^GUI: graphical user interface.

^i^SEAS: scientific exercise approach to scoliosis.

^j^GPR: global postural reeducation.

^k^RTT: Rett syndrome.

^l^AR: augmented reality.

^m^JIA: juvenile idiopathic arthritis.

#### Quality Assessment of Included Studies

Of the 21 included studies, 18 (86%) were assessed using the MMAT. Three studies were not evaluated: two were study protocols without results, and one was a feasibility study that focused solely on the accuracy of wearable sensor measurements. Although these studies could not be assessed using the MMAT, they provided valuable descriptions of digital interventions and were included in the synthesis.

The assessment results (Multimedia Appendix 4 [12,17,24,25,52-68]) showed that most studies (15/21, 71%) met only two or three MMAT criteria, primarily due to insufficient methodological detail. Only 2 (10%) studies met four criteria and were rated as high quality.

#### Sample Population

Of the 21 included studies, 15 (71%) specifically targeted patients with AIS, 4 (19%) studies included patients with AIS as part of a broader population, and 2 (10%) studies focused on patients with AIS with complications. Among the 20 studies that recruited participants, sample sizes ranged from 1 to 66, reflecting the relatively small scale of existing research. One additional study did not recruit any participants, as it focused solely on developing a noninvasive wearable smart vest designed to monitor spinal posture and movement for scoliosis rehabilitation, without conducting clinical or feasibility testing involving human participants. Additionally, four feasibility or pilot studies recruited nontarget populations, such as healthy adults beyond the typical AIS age range, to evaluate the feasibility or validity of digital interventions rather than clinical efficacy. For example, Caviedes et al [52] recruited 1 patient with scoliosis and 5 healthy adults (aged 22-64 years) to pilot a wearable sensor array, focusing on the technical validation of spine posture monitoring and real-time feedback during therapeutic spinal exercises. No clear age classification was derived from the studies.

#### Characteristics of Digital Intervention

The included studies used various types of digital technologies, which can be grouped into four categories (Table 4): digital content and platforms (videoconferencing, instructional videos, and mobile apps), VR and augmented reality (AR) systems, exergames, and wearable sensors. Most studies (17/21, 81%) provided multimodal feedback, with over half (15/21, 71%) combining visual and auditory feedback. A total of 2 (10%) studies provided haptic feedback: one used vibration to indicate incorrect posture, while the other applied lateral forces to simulate hippotherapy.

In total, 14 (67%) studies used digital content and platforms, making it the most common type of digital technology. These primarily supported synchronous or asynchronous home-based telerehabilitation, supplementing or partially replacing face-to-face therapy. A total of 7 (33%) studies used videoconferencing to enable physiotherapists to provide real-time guidance, supervision for home exercise, or feedback after sessions; 5 (24%) studies provided instructional videos for self-exercise, delivered through web-based platforms or DVDs; and 2 (10%) studies used mobile apps to facilitate communication between researchers, patients, and parents. These apps enabled researchers to monitor training, provide advice, and share health information through social messaging.

A total of 3 (14%) studies incorporated VR and AR into rehabilitation, often alongside other technologies, and 2 (10%) studies used VR systems: one involved physiotherapists managing training through a graphical user interface while patients engaged in hippotherapy simulations using an electromechanical device and a head-mounted display. The other used a head-mounted display, infrared tracking controllers, and a Wii Balance Board to create an immersive VR environment for posture correction. Only 1 (5%) study used an AR system with a mobile app and a handheld smart bar device to guide patient movements in real time through body imaging.

**Table 4:** Technology and intervention characteristics.

Characteristics				Studies (n=21), n (%)		Citations
**Technology type**						
	**Digital content and platforms (n=14, 67%)**					
		Videoconferencing	7 (33)		[25,57,58,63,66-68]	
		Instructional videos	5 (24)		[12,17,24,58,65]	
		Mobile apps	2 (10)		[56,60]	
	**VR^a^ and AR^b^ (n=3, 14%)**					
		VR system	2 (10)		[59,62]	
		AR system	1 (5)		[64]	
	Exergames			3 (14)		[53,55,61]
	Wearable sensors			2 (10)		[52,54]
**Feedback mode**						
	Visual and auditory			15 (71)		[12,17,24,25,52,57,58,61-68]
	Visual			2 (10)		[53,55]
	Auditory and haptic			1 (5)		[54]
	Visual and haptic			1 (5)		[59]
	Not specified			2 (10)		[56,60]
**Intervention duration**						
	6 months			8 (38)		[12,53,55-57,63,66,67]
	2 months			3 (14)		[24,58,60]
	5 months			2 (10)		[25,65]
	3 months			2 (10)		[17,68]
	Not specified			2 (10)		[59,62]
	N/A^c^			4 (19)		[52,54,61,64]
**Intervention location**						
	**Home and clinic setting**			8 (38)		[17,24,55,56,60,65,66,68]
		Initial clinic sessions followed by home exercise		4 (19)		[24,60,66,68]
		Home-based sessions with regular clinic visits		5 (24)		[17,24,55,56,65]
	Home			7 (33)		[12,25,53,57,58,63,67]
	Research setting			1 (5)		[59]
	School			1 (5)		[62]
	N/A			4 (19)		[52,54,61,64]
**Physiotherapist involvement**						
	Face-to-face			6 (29)		[17,24,55,59,62,65]
	Remote (synchronous)			4 (19)		[25,57,58,67]
	Remote (asynchronous)			3 (14)		[12,61,63]
	Face-to-face and remote (asynchronous)			2 (10)		[56,60]
	Face-to-face and remote (synchronous)			2 (10)		[66,68]
	Not specified			1 (5)		[53]
	Not reported			3 (14)		[52,54,64]
**Intervention outcome**						
	Cobb angle			12 (57)		[25,53,55-57,59,60,63,65-68]
	**Trunk asymmetry (n=9, 43%)**					
		ATR^d^	6 (29)		[17,56,57,60,66,68]	
		WRVAS^e^	2 (10)		[17,66]	
		ATSI/POTSI^f^	1 (5)		[65]	
	Quality of life (SRS-22^g^)			4 (19)		[12,17,56,60]
	Adherence			3 (14)		[12,55,58]
	Physical activity level			3 (14)		[12,63,68]
	Motor function			2 (10)		[24,63]
	Pain			2 (10)		[24,66]
	Respiratory function			2 (10)		[66,68]
	Other			11 (52)		[12,17,24,55-58,60,62,63,67]
	N/A			4 (19)		[52,54,61,64]

^a^VR: virtual reality.

^b^AR: augmented reality.

^c^Not applicable.

^d^ATR: angle of trunk rotation.

^e^WRVAS: Walter Reed Visual Assessment Scale.

^f^ATSI/POTSI: Anterior and Posterior Trunk Symmetry Indexes.

^g^SRS-22: Quality of Life questionnaire of the Scoliosis Research Society.

A total of 3 (14%) studies used exergames for rehabilitation. Two studies used game controllers to track limb and trunk movements, allowing patients to control game objects in real time, while another used an inertial measurement unit–based wearable system for posture detection. All exergames were 2D and allowed for remote supervision by recording exercise data.

In total, 2 (10%) studies used wearable sensors, such as inertial and stretch sensors embedded in clothing or harnesses, to monitor posture during unsupervised home exercises. These wearable systems also provided real-time feedback and enabled remote monitoring.

Intervention durations ranged from 2 to 6 months, with 6 months being the most common (8/21, 38%). Most interventions (15/21, 71%) were conducted at home, with 8 (38%) studies combining home and clinic settings. These studies followed two distinct intervention arrangements: initial clinic sessions followed by home exercises (4/21, 19%), and home-based sessions supplemented by regular clinic visits (5/21, 24%). Notably, one study used both arrangements in different study arms, assigning initial clinic sessions to the control group and regular clinic follow-ups to the intervention group. A total of 17 (81%) studies involved physiotherapists, delivering services through face-to-face (6/21, 29%), remote (7/21, 33%), or combined formats (4/21, 19%).

Over half of the studies (12/21, 57%) reported changes in the Cobb angle, with only 6 (29%) showing significant improvement. A total of 9 (43%) studies assessed trunk asymmetry using imaging parameters like the angle of trunk rotation and the Anterior and Posterior Trunk Symmetry Indexes, or questionnaires such as the Walter Reed Visual Assessment Scale. Less commonly reported outcomes included quality of life (4/21, 19%), adherence (3/21, 14%), physical activity level (3/21, 14%), motor function (2/21, 10%), pain (2/21, 10%), and respiratory function (2/21, 10%).

#### User Experience of the Digital Intervention

Only 3 of 21 (14%) studies [12,59,61] reported qualitative feedback on user experiences with digital interventions, which were included in the narrative synthesis. Interviews and open-ended questionnaires gathered subjective views from patients, parents, and physiotherapists, revealing four key themes. The raw qualitative data and corresponding themes are provided in Multimedia Appendix 5 [12,59,61].

#### Home Rehabilitation Support

Patients expressed a willingness to engage in home-based rehabilitation supported by digital technologies, highlighting its convenience over traditional clinic settings (“increase their interest in home-based training” [61] and “exercises could be done at home easily” [12]). Physiotherapists noted that digital tools helped monitor patients’ home training (“observe the patient’s home training state” [61]).

#### Engaging and User-Friendly Content

Engaging and user-friendly digital content was considered essential, especially for adolescents and children. Patients found digital interventions interesting (“exercises were interesting” [12] and “found the treatment enjoyable” [59]). However, some felt the interaction mechanism was inconvenient and needed improvement (“the interaction of SRA was a little inconvenient” [61]).

#### Social and Professional Support

Support from social networks and professionals enhanced the effectiveness of digital interventions, providing opportunities for patient socialization. Physiotherapists reported that remote supervision and feedback ensured that exercise programs were followed correctly (“prevent patients from poor self-control and poor training effect at home” [61]). Patients and parents also believed peer interaction boosted motivation and adherence (“competing or sharing with other participants” [12]).

#### Personalized Rehabilitation

Physiotherapists gave positive feedback on adjusting training content in real time based on patients’ physiological indicators (“judged the experience of working with the prototype favorably” [59]). However, patients noted low accuracy in movement recognition by some digital systems (“even if they made the corresponding action, SRA still estimated that his action was wrong” [61]), citing a lack of adaptability to individual patient needs. These findings underscore the importance of personalized rehabilitation tailored to each patient’s condition.

#### Theoretical Basis and BCTs

None of the 21 studies explicitly reported a theoretical foundation or specified the BCTs used. Instead, most interventions were designed based on clinical practice and experience, focusing on exercise regimens and outcomes. To address this, the Michie taxonomy was applied to code BCTs from intervention descriptions [35]. A total of 22 out of 93 possible BCTs were identified across the studies, spanning 11 of 16 BCT clusters (Table 5). Each study used between 2 and 9 BCTs (mean 6.1, SD 2.3; median 6, IQR 4.5-8.5). The most frequently used BCT cluster was “Shaping Knowledge” (19/21, 91%), followed by “Social Support” (16/21, 76%), “Antecedents” (16/21, 76%), “Goals and Planning” (15/21, 71%), “Repetition and Substitution” (15/21, 71%), and “Feedback and Monitoring” (14/21, 67%). Five BCT clusters, including “Regulation,” “Identity,” “Scheduled Consequences,” “Self-Belief,” and “Covert Learning,” were not identified in any study.

The most commonly used BCT was “Instruction on how to perform the behavior” (19/21, 91%), with most studies providing clear instructions for correct exercise behaviors. The next three BCTs were used in 71% (15/21) of studies: “Action Planning,” “Behavioral Practice or Rehearsal,” and “Social Support (Practical).” These studies developed detailed training plans specifying exercise duration and frequency, with practical support from physiotherapists or parents. Additionally, 67% (14/21) of studies used “Adding Objects to the Environment,” providing patients with devices to aid the exercise process.

The next five BCTs were used less frequently. “Feedback on behavior” (7/21, 33%) was delivered digitally to help patients improve their posture and movements. “Self-monitoring of behavior” (7/21, 33%) involved patients self-reporting exercise details at home. “Demonstration of the Behavior” (7/21, 33%) provided exercise examples through physiotherapists or videos. “Generalization of Target Behavior” (6/21, 29%) encouraged patients to apply correct postures to daily life activities. “Graded Tasks” (6/21, 29%) involved adjusting training difficulty and progressively increasing exercise frequency and complexity.

Some BCTs were rarely used. A total of 14% (3/21) of studies used “Problem Solving,” in which physiotherapists collaborated with patients and families to identify barriers to adherence and adjust training plans. “Habit Formation” (2/21, 10%) encouraged patients to incorporate repeated exercises into their daily routines. “Credible Source” (2/21, 10%) referred to physiotherapists providing persuasive guidance on disease knowledge and exercise instructions. “Restructuring the Physical Environment” (2/21, 10%) involved creating immersive virtual environments conducive to correct posture training. Additionally, eight other BCTs were used in only one study each.

**Table 5:** BCTs^a^ and BCT categories used in the included studies^b^.

BCT cluster and individual BCT		Studies (n=21), n (%)	Citations
**1. Goals and planning (n=15, 71%)**			
	1.1. Goal setting (behavior)	1 (5)	[63]
	1.2. Problem solving	3 (14)	[17,56,63]
	1.4. Action planning	15 (71)	[12,17,24,25,55-58,60,62,63,65-68]
**2. Feedback and monitoring (n=14, 67%)**			
	2.1. Monitoring of behavior by others without feedback	1 (5)	[60]
	2.2. Feedback on behavior	7 (33)	[52-55,61,62,64]
	2.3. Self-monitoring of behavior	7 (33)	[12,17,24,55,56,58,68]
	2.6. Biofeedback	1 (5)	[62]
**3. Social support (n=16, 76%)**			
	3.1. Social support (unspecified)	1 (5)	[12]
	3.2. Social support (practical)	15 (71)	[17,24,25,55-60,62,63,65-68]
**4. Shaping knowledge (n=19, 91%)**			
	4.1. Instruction on how to perform the behavior	19 (91)	[12,17,24,25,53,55-68]
**5. Natural consequences (n=1, 5%)**			
	5.1. Information about health consequences	1 (5)	[60]
**6. Comparison of behavior (n=7, 33%)**			
	6.1. Demonstration of the behavior	7 (33)	[12,17,24,58,63-65]
**7. Associations (n=1, 5%)**			
	7.1. Prompts or cues	1 (5)	[58]
**8. Repetition and substitution (n=15, 71%)**			
	8.1. Behavioral practice or rehearsal	15 (71)	[12,17,24,25,53,55-58,60,63,65-68]
	8.3. Habit formation	2 (10)	[12,66]
	8.6. Generalization of target behavior	6 (29)	[55,56,60,63,66,67]
	8.7. Graded tasks	6 (29)	[17,24,53,55,58,68]
**9. Comparison of outcomes (n=2, 10%)**			
	9.1. Credible source	2 (10)	[24,60]
**10. Reward and threat (n=2, 10%)**			
	10.1. Material incentive (behavior)	1 (5)	[60]
	10.3. Nonspecific reward	1 (5)	[62]
**12. Antecedents (n=16, 76%)**			
	12.1. Restructuring the physical environment	2 (10)	[59,62]
	12.5. Adding objects to the environment	14 (67)	[17,24,25,52-58,61,64-66]

^a^BCT: behavior change technique.

^b^BCTs not identified during the intervention coding process in this review were excluded from the table.

#### TDF Domains and the COM-B Model Mapping

The BCTs identified were linked to 7 TDF domains, corresponding to all six subcomponents of the COM-B model (Table 6). Each study used between 2 and 6 TDF domains (mean 4.5, SD 1.4; median 5, IQR 3-5.5) and targeted 2 to 5 COM-B model components (mean 4.1, SD 1.1; median 5, IQR 3-5). Of the 21 studies, 71% (15/21) of studies addressed “Capability,” “Opportunity,” and “Motivation” simultaneously, while the remaining 29% (6/21) focused only on “Capability” and “Opportunity.” Notably, the BCT “Credible Source” was not included in Table 6, as it could not be mapped to any specific TDF domain.

**Table 6:** TDF^a^ domains and COM-B^b^ model components mapping.

TDF domain	BCTs^c^	Frequency of use (n=21), n (%)	COM-B component
Knowledge	2.2. Feedback on behavior 2.6. Biofeedback 4.1. Instruction on how to perform the behavior 5.1. Information about health consequences	21 (100)	Psychological capability
Skills	2.1. Monitoring of behavior by others without feedback 6.1. Demonstration of the Behavior 8.1 Behavioral practice or rehearsal 8.3. Habit formation 8.6. Generalization of target behavior 8.7. Graded tasks	16 (76)	Physical capability
Environmental context and resources	7.1. Prompts or cues 12.1. Restructuring the physical environment 12.5. Adding objects to the environment	16 (76)	Physical opportunity
Social influences	3.1. Social support (unspecified) 3.2. Social support (practical)	16 (76)	Social opportunity
Goals	1.1. Goal setting (behavior) 1.4. Action planning	15 (71)	Reflective motivation
Behavioral regulation	1.2. Problem solving 2.3. Self-monitoring of behavior	8 (38)	Psychological capability
Reinforcement	10.1. Material incentive (behavior) 10.3. Nonspecific reward	2 (10)	Automatic motivation

^a^TDF: Theoretical Domains Framework.

^b^COM-B: capability, opportunity, motivation, and behavior.

^c^BCT: behavior change technique.

The most common TDF domain was “Knowledge,” used in all studies (21/21, 100%). By using BCTs such as “Instruction on how to perform the behavior” and “Feedback on behavior,” this mechanism aimed at enhancing patients’ cognitive understanding of the disease and rehabilitation, thereby strengthening their “Psychological Capability” and confidence.

Other common TDF domains included “Skills” (16/21, 76%), “Environmental Context and Resources” (16/21, 76%), “Social Influences” (16/21, 76%), and “Goals” (15/21, 71%). “Skills” were targeted using BCTs like “Behavioral Practice or Rehearsal,” improving patients’ “Physical Capability” to perform exercises effectively. “Environmental Context and Resources” were addressed through the BCT “Adding Objects to the Environment,” providing necessary resources and optimizing training environments to boost patients’ “Physical Opportunity” for rehabilitation. “Social Influences” were primarily facilitated through the BCT “Social Support (Practical),” offering emotional and practical support from physiotherapists and family members to bolster patients’ “Social Opportunity.” “Goals” were typically achieved through the BCT “Action Planning,” setting clear goals and specific plans to enhance patients’ “Reflective Motivation” toward achieving rehabilitation targets.

“Behavioral Regulation” appeared in 38% (8/21) of the studies, often achieved through BCTs such as “Self-monitoring of behavior” and “Problem Solving.” These techniques enabled patients to self-assess and adjust their training processes, enhancing their “Psychological Capability” to engage in and adhere to training plans. Finally, only 10% (2/21) of the studies used “Reinforcement,” using BCTS like “Material Incentive (Behavior)” and “Nonspecific Reward.” These reward mechanisms enhanced patients’ “Automatic Motivation,” encouraging sustained participation.

## Discussion

### Summary of Evidence

This scoping review summarizes the current literature on digital exercise interventions for patients with AIS, identifying the BCTs used and their potential mechanisms of action. A total of 21 studies were included, with most (15/21, 71%) published in the last 4 years, and nearly half (9/21, 43%) being feasibility or pilot studies. These findings suggest that this is a rapidly growing field with significant potential for future research.

The included studies varied in the exercise methods. While PSSE is widely recognized for scoliosis treatment, only half of the studies used PSSE methods, with the rest favoring GTE. This diversity may result from the current lack of high-quality evidence supporting the effectiveness of either PSSE or GTE for AIS, or demonstrating the superiority of one over the other [9,73]. Additionally, no clear relationship between exercise methods and digital technologies was found, as the choice of digital tools did not seem influenced by the type of exercises. Future research should explore the effectiveness of combining specific exercise methods with digital technologies and clarify their supportive role.

Digital technologies play several roles in AIS exercise rehabilitation, each offering distinct advantages and limitations. First, digital content and platforms have transformed traditional methods into telerehabilitation through internet communication, extending the reach of face-to-face rehabilitation. The rise of telerehabilitation, likely accelerated by the COVID-19 pandemic, offers low cost and broad accessibility, particularly benefiting patients in remote areas [49]. These technologies enable physiotherapists to supervise and guide home exercises either synchronously (eg, via videoconferencing) or asynchronously (eg, via instructional videos), thereby improving the feasibility of home rehabilitation [67]. Telerehabilitation has been shown to be a safe and effective complement to AIS treatment [67]. However, it also presents challenges, such as the absence of physical contact between physiotherapists and patients and reliance on stable internet connections [67,74]. Compared to fully remote programs, several studies suggest that a hybrid model combining web-based and offline approaches may better meet patient needs. For example, Dursun et al [68] found that continuing home rehabilitation after a clinical program enhances both its effectiveness and sustainability. Second, VR and AR have been applied as auxiliary tools in conventional rehabilitation but remain underexplored in exercise-based rehabilitation [75,76]. VR creates immersive simulated environments, while AR overlays virtual elements onto real-world scenes [77]. In the included studies, VR and AR were used alongside other devices, such as a handheld device [64], a force platform [62], or a mechatronic horse-riding simulator [59]. These technologies enhance immersion, maintain attention, and boost engagement [49,75]. For instance, Rösner et al [59] found that most participants preferred immersive VR environments, suggesting that VR systems could improve user experience. However, VR and AR systems tend to be expensive, limiting their use outside of research settings, and may cause side effects such as dizziness or visual fatigue [49,77]. Future studies should focus on developing cost-effective solutions and evaluating long-term effectiveness in home rehabilitation [76].

Third, exergames have shown benefits in exercise rehabilitation for conditions such as neurological disorders and childhood obesity [55,78]. Unlike commercial games, the exergames in the included studies were custom-developed and incorporated inertial measurement unit sensors [61] or game controllers [53,55] to capture real-time postural movements. Exergames complement traditional exercise rehabilitation by providing enjoyable, gamified experiences [78,79]. Their customized design also enables researchers to tailor content to individual needs [55,79]. Nonetheless, some studies reported challenges such as declining motivation, poor adherence, and an increased risk of adverse events [55,80]. For example, Wibmer et al [55] found that patients with AIS reduced their exercise frequency as interest in exergames waned, a finding consistent with other studies [81,82]. Finally, wearable sensors monitor posture to support independent exercise, partially replacing physiotherapists and enabling more complex interventions. Current studies focus on the technical aspects of posture monitoring, consistent with previous findings that physiotherapists consider body position tracking via digital tools a key priority for home rehabilitation [26]. Wearable sensors are well-suited for home use, offering real-time feedback and allowing physiotherapists to adjust programs remotely based on collected data, thereby facilitating personalized rehabilitation [83,84]. However, limitations include concerns about data privacy and limited adaptability in home environments [85]. Although wearable sensors have been widely studied in general telerehabilitation, this review highlights their underutilization in AIS rehabilitation, suggesting a promising direction for future research. Future studies should explore the integration of multiple digital technologies to develop more effective, comprehensive rehabilitation programs.

Current digital interventions lack sufficient focus on feedback design, despite the crucial role of multimodal feedback in helping patients accurately understand and perform movements [86]. Visual and haptic feedback, in particular, warrant further research. VR and AR technologies provide immersive visual feedback by overlaying virtual images onto real environments or creating fully virtual settings, which may reduce patients’ perception of physical pain during corrective exercises [86,87]. Haptic feedback offers direct and precise motion guidance, which is especially valuable in home-based rehabilitation without physiotherapist involvement. Andrade et al [67] noted that telerehabilitation lacks the direct physical contact of traditional therapy, limiting hands-on treatment by physiotherapists. Haptic feedback could address this limitation by guiding patients to control limb positions accurately, compensating for the shortcomings of telerehabilitation. Future research should delve deeper into feedback modality design in digital interventions to improve rehabilitation outcomes and patient experiences.

Our findings show that “Shaping Knowledge,” “Goals and Planning,” “Feedback and Monitoring,” and “Repetition and Substitution” are common BCT clusters, consistent with broader reviews [16,88]. Additionally, “Social Support” and “Antecedents” are frequently used, likely due to the need for auxiliary equipment (eg, yoga mats and resistance bands) and digital devices in scoliosis therapy, as well as the social support required to help patients cope with challenges [26]. Although “Reward and Threat” and “Natural Consequences” were rarely used, they have shown potential in other studies, such as interventions for musculoskeletal and pediatric patients [88,89]. A review of digital interventions for children and adolescents also highlights the role of “Reward Systems” and “Guidance” in enhancing cognition and motivating behavior change [90]. Despite identifying common BCTs, there is still insufficient evidence on the most effective ones and how to integrate them optimally, which requires further research [89].

This review identifies “Knowledge,” “Skills,” “Environmental Context and Resources,” “Social Influences,” and “Goals” as the primary TDF domains that may support exercise rehabilitation and influence patient behavior, consistent with previous studies highlighting their positive impact [91,92]. Mapping to the COM-B model reveals that most studies focus on “Capability” and “Opportunity,” with “Psychological Capability” and “Physical Capability” being the most frequently mentioned subcomponents. This is expected since exercise rehabilitation aims to improve health through physical activity and exercise, and “Capability” is essential for behavior to occur [93]. Successful exercise rehabilitation for patients with AIS relies on their understanding of exercises, ability to use digital tools, and physical skills. Additionally, “Physical Opportunity” and “Social Opportunity,” as external drivers of behavior, are widely mentioned. Digital tools create opportunities for exercise and social interaction by optimizing environments and facilitating remote interactions between physiotherapists, patients, and parents throughout the rehabilitation cycle [94,95].

Although “Motivation” is recognized as a key factor in rehabilitation, current research gives it insufficient attention, particularly regarding “Automatic Motivation,” which is rarely discussed [96]. Most studies focus on “Reflective Motivation” as an external driver, primarily using the BCT “Action Planning” to raise patients’ awareness of rehabilitation plans without exploring additional strategies. “Automatic Motivation,” an unconscious, intrinsic driving force, is crucial for long-term adherence, as it fosters autonomous patient engagement [93,97,98]. Although some studies have used digital technologies to increase engagement and stimulate enthusiasm, this often leads to only temporary motivation, as the novelty of the technology tends to diminish over time [55,81,82]. Previous research has begun to explore strategies, such as BCTs, to enhance automatic motivation. A qualitative study on survivors of stroke found that BCTs related to reward or incentive, such as material and social rewards, can encourage sustained physical activity by activating automatic motivational processes, which align with the TDF domain of Reinforcement [99]. Additionally, studies based on self-determination theory suggest that certain self-regulatory BCTs, such as self-monitoring and reviewing behavioral goals, may enhance intrinsic motivation for physical activity; however, their specific connection to automatic motivation remains unclear [100,101]. Therefore, future studies should further investigate how digital interventions can effectively strengthen automatic motivation through these strategies to support lasting behavioral change in patients with AIS.

### Recommendations for Future Research

Based on these findings, we recommend the following key features for developing digital exercise interventions to empower patients with AIS in rehabilitation.

#### Enhancing Social Interaction

Digital technologies can enhance interactions between patients, physiotherapists, parents, and peers through tools like videoconferencing. For example, future artificial intelligence technologies could create virtual physiotherapist avatars to provide continuous and real-time support. This would reduce the burden on health care resources and potentially improve patient experience. Additionally, digital tools can foster richer interactions through digital collaborative or competitive tasks, and by building digital communities, further boosting engagement and motivation.

#### Offering Personalized Interventions

Digital technologies enable personalized interventions tailored to individual health conditions, needs, and goals. This supports physiotherapists in making precise decisions. Personalization also motivates patients, such as by customizing avatars in exergames to match their preferences, making the exercises more enjoyable. Additionally, real-time monitoring allows dynamic adjustments to rehabilitation plans based on patient progress and psychological state.

#### Empowering Patients With Greater Autonomy

Involving patients in the development of digital interventions enhances their ownership and acceptance of rehabilitation plans. Digital interventions should also give patients more autonomy throughout the rehabilitation process. For example, visualizing exercise data helps patients track their progress, increasing satisfaction and a sense of accomplishment. When patients observe the results of their efforts, they are more likely to engage actively in exercises. Furthermore, allowing patients to self-manage aspects of their rehabilitation, such as scheduling exercise times and selecting content, helps maintain motivation.

### Strengths and Limitations

To our knowledge, this scoping review is the first to summarize the use of digital technologies in AIS exercise rehabilitation, highlighting a promising area for further research. Additionally, it is the first to identify and map BCTs in digital interventions to TDF domains and COM-B model components, providing insights into the theoretical foundations of these interventions. This approach bridges the gap between theory and practice, offering valuable guidance for health care professionals and researchers in developing effective digital interventions.

However, this review has several limitations. First, none of the studies explicitly reported the BCTs used, so BCT identification relied on intervention descriptions using the BCTTv1 and expert consensus [35,41,50,51]. This process may introduce subjectivity, potentially affecting the results. To mitigate this, two researchers independently coded the data and held regular discussions. Second, the broad inclusion criteria encompassed studies involving other types of patients with scoliosis, patients with AIS complications, or nontarget populations. While this approach increased the number and diversity of studies analyzed, it also introduced heterogeneity in the study populations, which may limit the generalizability and clinical applicability of the findings to typical adolescent patients with AIS. Third, the varied roles of digital technologies added coding complexity, making it difficult to isolate their independent effects from other intervention components. Fourth, more than half of the included studies were feasibility or pilot studies, study protocols, or case studies, representing research types characterized by limited methodological rigor, incomplete intervention outcomes, or low generalizability. This prevalence inevitably reduces the overall robustness and applicability of the synthesized findings. Additionally, restricting the review to English-language studies may have affected its comprehensiveness. Finally, we were unable to retrieve the full text of 15 potentially eligible studies, as they were not available in databases or web-based sources. The exclusion of these studies may have introduced selection bias.

### Implications for Research and Practice

Future research should follow standardized reporting guidelines, such as CONSORT (Consolidated Standards of Reporting Trials) and TIDieR, to ensure detailed descriptions of digital interventions, including the content and implementation procedures. Additionally, there is a need to establish consistent definitions and classification frameworks for digital technologies to standardize the reporting of digital components across studies. This will enhance transparency, reproducibility, and comparability between interventions [48,102]. Furthermore, an initial theoretical understanding is essential for the design and evaluation of complex interventions, particularly those targeting behavior change [23,103]. However, none of the included studies reported a theoretical basis. Future research should explicitly report behavior change strategies, including the underlying theories and common taxonomies (eg, BCTTv1), to clarify intervention mechanisms and support the development of effective digital interventions [16,104]. Finally, a more comprehensive evaluation of digital interventions is needed. Future studies should incorporate long-term follow-up and adopt standardized outcome measures (eg, Cobb angle) to assess effectiveness. In addition, more in-depth qualitative research is required to explore user experiences, assess usability and home applicability, and inform future intervention design.

Additionally, due to limited evidence regarding how digital tools are practically used in real-world settings, designing and implementing digital interventions that effectively maintain adherence remains challenging. This review offers actionable implications for clinical practice. Physiotherapists and clinicians may consider using digital technologies as a complement to traditional interventions. When selecting appropriate technologies, it is important to account for their advantages and limitations, rehabilitation goals, and patient characteristics. For home-based rehabilitation, integrating digital interventions with regular in-person follow-ups is recommended. Moreover, incorporating behavior change elements into the intervention can help achieve desired target behaviors, such as enhancing engagement through goal setting. Finally, this review highlights key factors for empowering patients in digital interventions. These factors are linked to enhancing motivation for exercise adherence and can thus inform the design of conventional interventions as well.

### Conclusions

Digital technologies hold significant potential in AIS exercise rehabilitation, transforming traditional approaches by enhancing accessibility, patient engagement, and personalization. However, current interventions lack a strong theoretical foundation and explicit behavior change strategies, particularly in sustaining long-term patient motivation. To fully realize the benefits of digital interventions, future research should integrate behavior change theories and techniques to develop evidence-based, theory-driven digital solutions. These interventions should provide effective and sustainable rehabilitation options, improve long-term outcomes, and enhance the user experience.

## Appendix

Multimedia Appendix 1 PRISMA-ScR Checklist.

Multimedia Appendix 2 Search strategy.

Multimedia Appendix 3 Data extraction table.

Multimedia Appendix 4 Mixed Methods Appraisal Tool (MMAT) quality assessment.

Multimedia Appendix 5 Raw qualitative data and themes of user experience.

## Abbreviations

AIS: adolescent idiopathic scoliosis
AR: augmented reality
BCT: behavior change technique
BCTTv1: Behavior Change Technique Taxonomy v1
COM-B: capability, opportunity, motivation, and behavior
CONSORT: Consolidated Standards of Reporting Trials
GTE: general therapeutic exercise
MMAT: Mixed Methods Appraisal Tool
PRISMA-ScR: Preferred Reporting Items for Systematic Reviews and Meta-Analyses Extension for Scoping Reviews
PSSE: physiotherapeutic scoliosis–specific exercises
RCT: randomized controlled trial
TDF: Theoretical Domains Framework
TIDieR: Template for Intervention Description and Replication
VR: virtual reality
