# Supplementary material for: Behavior Change Strategies in Digital Exercise Interventions for Adolescent Idiopathic Scoliosis: Scoping Review
Source: J Med Internet Res. 2025 Sep 16;27:e66981. doi: 10.2196/66981 (PMC12485258; doi:10.2196/66981)
Supplement: Multimedia Appendix 2 [file jmir_v27i1e66981_app2.doc]

## Searches in PubMed

Search Date: April 4, 2024

| # | Searches | Results |
| --- | --- | --- |
| S1 | ("Scoliosis"[Mesh]) OR ((((Scolioses) OR (Idiopathic Scoliosis)) OR (Adolescent Idiopathic Scoliosis)) OR (Juvenile Idiopathic Scoliosis)) | 31,636 |
| S2 | ("Exercise Therapy"[Mesh]) OR (((((((((((((((((((Remedial Exercise*) OR (Exercise Therapies)) OR (Rehabilitation Exercise*)) OR (Exercise*)) OR (Training)) OR (Sport*)) OR (Conservative Treatment*)) OR (Conservative Intervention*)) OR (Physiothera*)) OR (Physical Activit*)) OR (Physical Therap*)) OR (Schroth)) OR (SEAS)) OR (DoboMed)) OR (Side Shift)) OR (Lyon)) OR (BSPTS)) OR (FITS)) OR (Stabilization)) | 5,214,967 |
| S3 | ("Digital Health"[Mesh]) OR ((((((((((Digital Health Technolog*) OR (Digital Intervention*)) OR (Digital)) OR (Wearable Device*)) OR (Wearable Sensor*)) OR (Exergaming*)) OR (Exergame*)) OR (Virtual Reality)) OR (Video Game*)) OR (Gamification)) | 784,839 |
| S4 | ("Telemedicine"[Mesh]) OR ((((((Telehealth) OR (eHealth)) OR (Mobile Health)) OR (mHealth)) OR (Telenursing)) OR (Telerehabilitation)) | 124,461 |
| S5 | ("Mobile Applications"[Mesh]) OR ((((((((((((((Mobile Application) OR (App)) OR (Application*)) OR (Smartphone)) OR (Smart-Phone)) OR (Telephone)) OR (Cellphone)) OR (Mobile)) OR (Email)) OR (E-Mail)) OR (Tablet)) OR (Cell)) OR (Computer-Assisted)) OR (Computer)) | 11,567,022 |
| S6 | ("Internet-Based Intervention"[Mesh]) OR (((((Web) OR (Website)) OR (Online)) OR (Internet)) OR (Social Media)) | 677,857 |
| S7 | S3 OR S4 OR S5 OR S6 | 12,194,610 |
| S8 | S1 AND S2 AND S7 | 1,338 |

## Searches in Web of Science

Search Date: April 4, 2024

| # | Searches | Results |
| --- | --- | --- |
| S1 | Scoliosis (Topic) or Scolioses (Topic) or Idiopathic Scoliosis (Topic) or Adolescent Idiopathic Scoliosis (Topic) or Juvenile Idiopathic Scoliosis (Topic) | 48,192 |
| S2 | Exercise Therapy (Topic) or Remedial Exercise* (Topic) or Exercise Therapies (Topic) or Rehabilitation Exercise* (Topic) or Exercise* (Topic) or Training (Topic) or Sport* (Topic) or Conservative Treatment* (Topic) or Conservative Intervention* (Topic) or Physiothera* (Topic) or Physical Activit* (Topic) or Physical Therap* (Topic) or Schroth (Topic) or SEAS (Topic) or DoboMed (Topic) or Side Shift (Topic) or Lyon (Topic) or BSPTS (Topic) or FITS (Topic) or Stabilization (Topic) | 12,013,987 |
| S3 | Digital Health (Topic) or Digital Health Technolog* (Topic) or Digital Intervention* (Topic) or Digital (Topic) or Wearable Device* (Topic) or Wearable Sensor* (Topic) or Exergaming* (Topic) or Exergame* (Topic) or Virtual Reality (Topic) or Video Game* (Topic) or Gamification (Topic) | 4,336,044 |
| S4 | Telemedicine (Topic) or Telehealth (Topic) or eHealth (Topic) or Mobile Health (Topic) or mHealth (Topic) or Telenursing (Topic) or Telerehabilitation (Topic) | 255,833 |
| S5 | Mobile Applications (Topic) or Mobile Application (Topic) or App (Topic) or Application* (Topic) or Smartphone (Topic) or Smart-Phone (Topic) or Telephone (Topic) and Cellphone (Topic) and Mobile (Topic) and Email (Topic) and E-Mail (Topic) and Tablet (Topic) and Cell (Topic) and Computer-Assisted (Topic) and Computer (Topic) | 16,062,558 |
| S6 | Internet-Based Intervention (Topic) or Web (Topic) or Website (Topic) or Online (Topic) or Internet (Topic) or Social Media (Topic) | 4,405,125 |
| S7 | #6 OR #5 OR #4 OR #3 | 22,595,535 |
| S8 | #1 AND #2 AND #7 | 817 |

## Searches in Embase

Search Date: April 4, 2024

| # | Searches | Results |
| --- | --- | --- |
| 1 | 'scoliosis'/exp | 44,858 |
| 2 | scolioses:ab,ti OR 'idiopathic scoliosis':ab,ti OR 'adolescent idiopathic scoliosis':ab,ti OR 'juvenile idiopathic scoliosis':ab,ti | 11,588 |
| 3 | #1 OR #2 | 45,198 |
| 4 | 'kinesiotherapy'/exp | 107,519 |
| 5 | 'exercise therapy':ab,ti OR 'remedial exercise*':ab,ti OR 'exercise therapies':ab,ti OR 'rehabilitation exercise*':ab,ti OR exercise*:ab,ti OR training:ab,ti OR sport*:ab,ti OR 'conservative treatment*':ab,ti OR 'conservative intervention*':ab,ti OR physiothera*:ab,ti OR 'physical activit*':ab,ti OR 'physical therap*':ab,ti OR schroth:ab,ti OR seas:ab,ti OR dobomed:ab,ti OR 'side shift':ab,ti OR lyon:ab,ti OR bspts:ab,ti OR fits:ab,ti OR stabilization:ab,ti | 1,709,919 |
| 6 | #4 OR #5 | 1,747,246 |
| 7 | 'digital health'/exp | 690 |
| 8 | 'digital health technolog*':ab,ti OR 'digital intervention*':ab,ti OR digital:ab,ti OR 'wearable device*':ab,ti OR 'wearable sensor*':ab,ti OR exergaming*:ab,ti OR exergame*:ab,ti OR 'virtual reality':ab,ti OR 'video game*':ab,ti OR gamification:ab,ti | 276,988 |
| 9 | #7 OR #8 | 277,096 |
| 10 | 'telemedicine'/exp | 75,591 |
| 11 | telehealth:ab,ti OR ehealth:ab,ti OR 'mobile health':ab,ti OR mhealth:ab,ti OR telenursing:ab,ti OR telerehabilitation:ab,ti | 37,772 |
| 12 | #10 OR #11 | 99,343 |
| 13 | 'mobile application'/exp | 27,824 |
| 14 | 'mobile applications':ab,ti OR app:ab,ti OR application*:ab,ti OR smartphone:ab,ti OR 'smart phone':ab,ti OR telephone:ab,ti OR cellphone:ab,ti OR mobile:ab,ti OR email:ab,ti OR 'e mail':ab,ti OR tablet:ab,ti OR cell:ab,ti OR 'computer assisted':ab,ti OR computer:ab,ti | 7,483,804 |
| 15 | #13 OR #14 | 7,489,155 |
| 16 | 'web-based intervention'/exp | 3,241 |
| 17 | 'internet-based intervention':ab,ti OR web:ab,ti OR website:ab,ti OR online:ab,ti OR internet:ab,ti OR 'social media':ab,ti | 683,352 |
| 18 | #16 OR #17 | 683,821 |
| 19 | #9 OR #12 OR #15 OR #18 | 8,253,384 |
| 20 | #3 AND #6 AND #19 | 455 |

## Searches in Cochrane Library

Search Date: April 4, 2024

| # | Searches | Results |
| --- | --- | --- |
| 1 | MeSH descriptor: [Scoliosis] explode all trees | 725 |
| 2 | (Scolioses or Idiopathic Scoliosis or Adolescent Idiopathic Scoliosis or Juvenile Idiopathic Scoliosis):ti,ab,kw | 936 |
| 3 | #1 or #2 | 1196 |
| 4 | MeSH descriptor: [Exercise Therapy] explode all trees | 21620 |
| 5 | (Remedial Exercise* or Exercise Therapies or Rehabilitation Exercise* or Exercise* or Training or Sport* or Conservative Treatment* or Conservative Intervention* or Physiothera* or Physical Activit* or Physical Therap* or Schroth or SEAS or DoboMed or Side Shift or Lyon or BSPTS or FITS or Stabilization):ti,ab,kw | 371312 |
| 6 | #4 or #5 | 371369 |
| 7 | MeSH descriptor: [Digital Health] explode all trees | 8 |
| 8 | (Digital Health Technolog* or Digital Intervention* or Digital or Wearable Device* or Wearable Sensor* or Exergaming* or Exergame* or Virtual Reality or Video Game* or Gamification):ti,ab,kw | 38202 |
| 9 | #7 or #8 | 38202 |
| 10 | MeSH descriptor: [Telemedicine] explode all trees | 4807 |
| 11 | (Telehealth or eHealth or Mobile Health or mHealth or Telenursing or Telerehabilitation):ti,ab,kw | 23220 |
| 12 | #10 or #11 | 25575 |
| 13 | MeSH descriptor: [Mobile Applications] explode all trees | 1941 |
| 14 | (Mobile Application or App or Application* or Smartphone or Smart-Phone or Telephone or Cellphone or Mobile or Email or E-Mail or Tablet or Cell or Computer-Assisted or Computer):ti,ab,kw | 423137 |
| 15 | #13 or #14 | 423137 |
| 16 | MeSH descriptor: [Internet-Based Intervention] explode all trees | 650 |
| 17 | (Web or Website or Online or Internet or Social Media):ti,ab,kw | 54350 |
| 18 | #16 or #17 | 54350 |
| 19 | #9 or #12 or #15 or #18 | 480678 |
| 20 | #3 and #6 and #19 | 120 |

## Searches in CINAHL

Search Date: April 4, 2024

| S | Searches | Search modes | Results |
| --- | --- | --- | --- |
| 1 | (MH "Scoliosis") | Boolean/Phrase | 7,009 |
| 2 | SU Scolioses OR SU Idiopathic Scoliosis OR SU Adolescent Idiopathic Scoliosis OR SU Juvenile Idiopathic Scoliosis | Boolean/Phrase | 1,217 |
| 3 | S1 OR S2 | Boolean/Phrase | 8,125 |
| 4 | (MH "Therapeutic Exercise") | Boolean/Phrase | 27,823 |
| 5 | SU Exercise Therapy OR SU Exercise Therapy OR SU Remedial Exercise* OR SU Exercise Therapies OR SU Rehabilitation Exercise* OR SU Exercise* OR SU Training OR SU Sport* OR SU Conservative Treatment* OR SU Conservative Intervention* OR SU Physiothera* OR SU Physical Activit* OR SU Physical Therap* OR SU Schroth OR SU SEAS OR SU DoboMed OR SU Side Shift OR SU Lyon OR SU BSPTS OR SU FITS OR SU Stabilization | Boolean/Phrase | 331,996 |
| 6 | S4 OR S5 | Boolean/Phrase | 331,996 |
| 7 | (MH "Digital Health") | Boolean/Phrase | 1,513 |
| 8 | SU Digital Health Technolog* OR SU Digital Intervention* OR SU Digital OR SU Wearable Device* OR SU Wearable Sensor* OR SU Exergaming* OR SU Exergame* OR SU Virtual Reality OR SU Video Game* OR SU Gamification | Boolean/Phrase | 31,081 |
| 9 | S7 OR S8 | Boolean/Phrase | 31,081 |
| 10 | (MH "Telemedicine") | Boolean/Phrase | 16,398 |
| 11 | SU Telehealth OR SU eHealth OR SU Mobile Health OR SU mHealth OR SU Telenursing OR SU Telerehabilitation | Boolean/Phrase | 19,861 |
| 12 | S10 OR S11 | Boolean/Phrase | 35,301 |
| 13 | (MH "Mobile Applications") | Boolean/Phrase | 12,699 |
| 14 | SU Mobile Application OR SU App OR SU Application* OR SU Smartphone OR SU Smart-Phone OR SU Telephone OR SU Cellphone OR SU Mobile OR SU Email OR SU E-Mail OR SU Tablet OR SU Cell OR SU Computer-Assisted OR SU Computer | Boolean/Phrase | 434,133 |
| 15 | S13 OR S14 | Boolean/Phrase | 434,133 |
| 16 | (MH "Internet-Based Intervention") | Boolean/Phrase | 1,204 |
| 17 | SU Web OR SU Website OR SU Online OR SU Internet OR SU Social Media | Boolean/Phrase | 162,517 |
| 18 | S16 OR S17 | Boolean/Phrase | 162,517 |
| 19 | S9 OR S12 OR S15 OR S18 | Boolean/Phrase | 621,799 |
| 20 | S3 AND S6 AND S19 | Boolean/Phrase | 24 |
