# Supplementary material for: Behavior Change Strategies in Digital Exercise Interventions for Adolescent Idiopathic Scoliosis: Scoping Review
Source: J Med Internet Res. 2025 Sep 16;27:e66981. doi: 10.2196/66981 (PMC12485258; doi:10.2196/66981)
Supplement: Multimedia Appendix 4 [file jmir_v27i1e66981_app4.docx]

# MMAT quality assessment

| **Study** | **Type of study** | **Screening questions** | | **Methodological quality criteria** | | | | | | | | | | | | | | | **Criteria met** |
| --- | --- | --- | --- | --- | --- | --- | --- | --- | --- | --- | --- | --- | --- | --- | --- | --- | --- | --- | --- |
|  |  | **S1** | **S2** | **2.1** | **2.2** | **2.3** | **2.4** | **2.5** | **3.1** | **3.2** | **3.3** | **3.4** | **3.5** | **4.1** | **4.2** | **4.3** | **4.4** | **4.5** |  |
| Feistritzer-Gröbl et al. [53] | Quantitative descriptive | 1 | 1 |  |  |  |  |  |  |  |  |  |  | 0 | 0 | 1 | 0 | 2 | 1 |
| Sardini et al. [54] | Feasibility Study | 1 | 1 |  |  |  |  |  |  |  |  |  |  |  |  |  |  |  | N/A |
| Zapata et al. [24] | Quantitative randomized controlled (trials) | 1 | 1 | 1 | 1 | 0 | 1 | 0 |  |  |  |  |  |  |  |  |  |  | 3 |
| Wibmer et al. [55] | Quantitative descriptive | 1 | 1 |  |  |  |  |  |  |  |  |  |  | 1 | 0 | 1 | 1 | 1 | 4 |
| Liang et al. [56] | Study protocol | 1 | 0 |  |  |  |  |  |  |  |  |  |  |  |  |  |  |  | N/A |
| Caviedes et al. [52] | Quantitative descriptive | 1 | 1 |  |  |  |  |  |  |  |  |  |  | 0 | 0 | 1 | 0 | 1 | 2 |
| Cozeta Anca et al. [57] | Quantitative non-randomized | 1 | 1 |  |  |  |  |  | 1 | 1 | 0 | 0 | 2 |  |  |  |  |  | 2 |
| Fishman. [25] | Quantitative non-randomized | 1 | 1 |  |  |  |  |  | 1 | 1 | 0 | 0 | 2 |  |  |  |  |  | 2 |
| Lau et al. [12] | Quantitative randomized controlled (trials) | 1 | 1 | 1 | 1 | 1 | 2 | 0 |  |  |  |  |  |  |  |  |  |  | 3 |
| Marin et al. [58] | Quantitative non-randomized | 1 | 1 |  |  |  |  |  | 1 | 1 | 1 | 0 | 2 |  |  |  |  |  | 3 |
| Rösner et al. [59] | Quantitative descriptive | 1 | 1 |  |  |  |  |  |  |  |  |  |  | 0 | 0 | 1 | 1 | 1 | 3 |
| Wang et al. [60] | Study protocol | 1 | 0 |  |  |  |  |  |  |  |  |  |  |  |  |  |  |  | N/A |
| Li et al. [61] | Quantitative descriptive | 1 | 1 |  |  |  |  |  |  |  |  |  |  | 0 | 0 | 1 | 0 | 1 | 2 |
| Moraes et al. [62] | Quantitative non-randomized | 1 | 1 |  |  |  |  |  | 0 | 1 | 0 | 0 | 1 |  |  |  |  |  | 2 |
| Romano et al. [63] | Quantitative non-randomized | 1 | 1 |  |  |  |  |  | 1 | 1 | 0 | 0 | 1 |  |  |  |  |  | 3 |
| Nam et al. [64] | Quantitative descriptive | 1 | 1 |  |  |  |  |  |  |  |  |  |  | 0 | 1 | 1 | 0 | 1 | 3 |
| Vagner and Bendikova. [65] | Quantitative descriptive | 1 | 1 |  |  |  |  |  |  |  |  |  |  | 0 | 0 | 1 | 0 | 1 | 2 |
| Kisa et al. [66] | Quantitative randomized controlled (trials) | 1 | 1 | 1 | 1 | 0 | 1 | 2 |  |  |  |  |  |  |  |  |  |  | 3 |
| Mantelatto Andrade et al. [67] | Quantitative non-randomized | 1 | 1 |  |  |  |  |  | 1 | 1 | 1 | 0 | 2 |  |  |  |  |  | 3 |
| Manzak Dursun et al. [68] | Quantitative randomized controlled (trials) | 1 | 1 | 0 | 1 | 0 | 1 | 2 |  |  |  |  |  |  |  |  |  |  | 2 |
| Tombak et al. [17] | Quantitative randomized controlled (trials) | 1 | 1 | 1 | 1 | 0 | 1 | 1 |  |  |  |  |  |  |  |  |  |  | 4 |
| **1 = yes; 0 = no; 2 = can't tell** | | | | | | | | | | | | | | | | | | | |
