# Supplementary material for: Behavior Change Strategies in Digital Exercise Interventions for Adolescent Idiopathic Scoliosis: Scoping Review
Source: J Med Internet Res. 2025 Sep 16;27:e66981. doi: 10.2196/66981 (PMC12485258; doi:10.2196/66981)
Supplement: Multimedia Appendix 5 [file jmir_v27i1e66981_app5.docx]

# Raw qualitative data and themes of user experience

| **Study** | **Digital form** | **Qualitative methods and Participants** | **Qualitative data** | **Themes** |
| --- | --- | --- | --- | --- |
| Lau RW et al [12], 2021 | Web-based software providing exercise videos | Feedback Questionnaires; Patients and parents. | “exercises could be done at home easily” (Patients) | Home rehabilitation support |
|  |  |  | “the accelerometer was... convenient to use” (Patients) | Home rehabilitation support |
|  |  |  | “the exercises were interesting” (Patients) | Engaging and user-friendly content |
|  |  |  | “compliance could be enhanced by... competing/ sharing with other subjects...” (Patients and parents) | Social and professional support |
| Rösner D et al [59], 2021 | VR system comprising mechatronic device, therapist GUI, and synchronized visualization | Follow-up interviews; Patients and physiotherapists. | “they would like to try a similar type of therapy again... found the treatment enjoyable” (Patients) | Engaging and user-friendly content |
|  |  |  | “judged the experience... favorably, noting that the GUI offers an efficient way of creating a force progression which matches an individual patient’s breathing rhythm” (Physiotherapists) | Personalized rehabilitation |
| Li J et al [61], 2022 | Wearable system with training games | Follow-up interviews; Students and physiotherapists. | “could increase their interest in home-based training” (Students) | Engaging and user-friendly content |
|  |  |  | “through SRA can observe the patient at home training state, in order to prevent patient from poor self-control and poor training effect at home” (Physiotherapists) | Home rehabilitation support; Social and professional support |
|  |  |  | “even if they made the corresponding action, SRA still estimate that his action was wrong” (Students) | Personalized rehabilitation |
|  |  |  | “the game might not directly display their spinal state, and the interaction of SRA was a little inconvenient” (Students) | Engaging and user-friendly content |
